# The association between treatment burden and quality of life in older adults with multimorbidity: the mediating role of personal mastery and the moderating role of social support—a cross-sectional study in China

**DOI:** 10.3389/fpubh.2026.1859774

**Published:** 2026-08-03

**Authors:** Yanmin Weng, Di Wang, Qian Ma, Wen Ye, Pengcheng Wang, Xuerui Wang, Yin Zhang, Ping Zhang, Yingying Xu

**Affiliations:** 1 Department of Vascular Surgery, Nanjing Drum Tower Hospital, The Affiliated Hospital of Nanjing University Medical School, Nanjing, China; 2 Department of Nursing, Nanjing Drum Tower Hospital, The Affiliated Hospital of Nanjing University Medical School, Nanjing, China; 3 Jiangsu Longtan Prison, Nanjing, China; 4 Department of General Surgery, Nanjing Drum Tower Hospital, The Affiliated Hospital of Nanjing University Medical School, Nanjing, China; 5 Department of Urology, Nanjing Drum Tower Hospital, The Affiliated Hospital of Nanjing University Medical School, Nanjing, China; 6 Department of Cardiothoracic Surgery, Nanjing Drum Tower Hospital, The Affiliated Hospital of Nanjing University Medical School, Nanjing, China; 7 Department of General Surgery, Nanjing Drum Tower Hospital, The Affiliated Hospital of Nanjing University Medical School, Nanjing, China

**Keywords:** health-related quality of life, multimorbidity, older adults, personal mastery, social support, treatment burden

## Abstract

**Background:**

Treatment burden is associated with health-related quality of life (HRQoL) in multimorbidity, but its psychological and social correlates in older adults remain unclear. This study examined whether personal mastery was statistically linked to the association between treatment burden and HRQoL and whether social support moderated the association between treatment burden and personal mastery.

**Methods:**

A cross-sectional survey was conducted among 470 outpatients aged 60 years and older with multimorbidity in Jiangsu Province, China. Treatment burden, personal mastery, social support, and HRQoL were assessed using validated scales. Pearson correlations and PROCESS Model 7 with 5,000 bootstrap resamples were used, adjusting for age, BMI, sex, income, and education.

**Results:**

Treatment burden was negatively associated with personal mastery (*b* = −0.1185, *p* < 0.001), and the treatment burden × social support interaction was significant (*b* = 0.0056, *p* < 0.001). Personal mastery was positively associated with PCS (*b* = 0.4064, *p* = 0.005) and MCS (*b* = 0.3894, *p* = 0.028). Treatment burden showed a negative direct association with PCS (*b* = −0.1417, *p* < 0.001) but a positive direct association with MCS (*b* = 0.3639, *p* < 0.001). The indices of moderated mediation were significant for PCS (index = 0.0023, 95% CI [0.0006, 0.0042]) and MCS (index = 0.0022, 95% CI [0.0002, 0.0047]).

**Conclusion:**

Greater treatment burden was associated with poorer physical HRQoL and showed negative indirect associations with physical and mental HRQoL through lower personal mastery. These indirect associations were weaker at higher social support.

## 1 Introduction

Population ageing has made multimorbidity an increasingly important public health challenge worldwide. Multimorbidity is commonly defined as the coexistence of two or more chronic conditions in the same individual (1). In China, rapid population ageing has been accompanied by a growing burden of multimorbidity among older adults. Adults aged 60 years and older accounted for 18.70% of the national population in 2020, and evidence from the China Health and Retirement Longitudinal Study showed that the prevalence of multimorbidity among Chinese older adults increased from 46.16% in 2011 to 57.50% in 2018 (2, 3). Older adults with multimorbidity often experience higher healthcare use, functional decline, and poorer health-related quality of life (HRQoL), making the improvement of HRQoL in this population an important clinical and public health priority.

Previous research has shown that the association between multimorbidity and quality of life cannot be explained by disease count alone (4). Older adults often need to manage complex medication regimens, attend repeated appointments, monitor symptoms, make lifestyle adjustments, and coordinate care across providers (5). These healthcare-related demands may themselves become burdensome. Treatment burden refers to the workload that healthcare places on patients and the effects of that workload on their functioning and well-being (6). A recent integrative review suggests that treatment burden is common among people with multimorbidity and is associated with poorer patient-reported outcomes, including reduced quality of life and greater difficulty with self-management (5).

However, the association between treatment burden and HRQoL may differ across older adults because it is likely shaped by individual psychological resources. One theoretically plausible explanation is that treatment burden is associated with HRQoL through personal mastery, a general sense of control over important life circumstances and perceived ability to manage life demands (7, 8). The cumulative complexity model suggests that patient outcomes depend on the balance between the workload imposed by healthcare and the capacity available to patients to accomplish that workload (9). When healthcare-related tasks accumulate and exceed older adults’ perceived capacity, illness management may be experienced as less controllable and more externally imposed, which may be reflected in lower personal mastery (8). Thus, reduced personal mastery may represent a statistical indirect association linking treatment burden with poorer HRQoL.

At the same time, the association between treatment burden and personal mastery may depend on the social context in which older adults manage chronic illness. According to the stress-buffering hypothesis, social support can protect individuals from the harmful effects of stressors by providing emotional, practical, and informational resources (10). In older adults, social support has been linked to better health and well-being, suggesting that it may weaken the negative association between treatment burden and personal mastery and may be relevant to HRQoL (11, 12).

Despite growing interest in treatment burden, several gaps remain. While prior research has explored the association between treatment burden and HRQoL, less is known about the psychological correlates and social contextual conditions involved in this association, especially among older adults with multimorbidity in China. In particular, few studies have simultaneously examined the statistical indirect role of a psychological resource and the moderating role of a social resource in this association.

**Figure 1:**
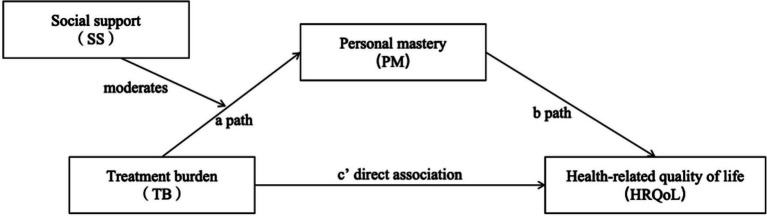
Conceptual diagram of the hypothesized moderated mediation model.

Accordingly, this study investigated older adults with multimorbidity in Jiangsu Province, China. The hypothesized moderated mediation model is shown in Figure 1. We examined whether personal mastery was statistically linked to the association between treatment burden and HRQoL and whether social support moderated the association between treatment burden and personal mastery. We hypothesized that higher treatment burden would be associated with poorer HRQoL, that personal mastery would statistically account for part of this association, and that social support would moderate the association between treatment burden and personal mastery, such that the indirect association between treatment burden and HRQoL through personal mastery would vary across levels of social support.

## 2 Methods

### 2.1 Study design and participants

This cross-sectional study was conducted in Jiangsu Province, East China, from March 2024 to December 2025. Participants were recruited from the outpatient chronic disease clinic of Nanjing Drum Tower Hospital, the Affiliated Hospital of Nanjing University Medical School, a large tertiary hospital in Jiangsu Province.

The sample size was estimated using G*Power 3.1 software. Because the planned moderated mediation analysis was regression-based, linear multiple regression with fixed-model R^2^ increase was selected for sample-size estimation. The interaction term between treatment burden and social support was treated as the focal tested predictor. Considering that interaction effects in observational moderated mediation studies are often small, a small effect size was assumed according to Cohen’s convention (*f*^2^ = 0.02) (13). With *α* = 0.05, statistical power = 0.80, one tested predictor, and eight total predictors in the largest regression equation, the minimum required valid sample size was 395. To account for potential nonresponse and invalid questionnaires during the survey process, an estimated nonresponse or invalid questionnaire rate of 20% was considered, resulting in a target sample size of at least 494 participants. Ultimately, 500 questionnaires were distributed, and 470 valid questionnaires were returned, yielding a valid response rate of 94.0%. Because this was a cross-sectional survey, no follow-up was involved. The remaining 30 questionnaires were excluded because of invalid or incomplete responses. The final analytic sample exceeded the minimum required valid sample size and was considered adequate for the planned moderated mediation analyses.

Eligible patients were consecutively approached in the outpatient clinic and invited to participate. The inclusion criteria were as follows: (1) age ≥60 years; (2) diagnosis of at least two chronic diseases, with a duration of at least 1 year for each condition; and (3) ability and willingness to provide informed consent and complete the survey. Multimorbidity was ascertained primarily based on outpatient medical records and supplemented by participant-reported disease history using a predefined chronic disease list. In the present study, chronic conditions included the index vascular disease and other physician-diagnosed chronic diseases, such as hypertension, diabetes mellitus, coronary heart disease, cerebrovascular disease, hyperlipidemia, chronic kidney disease/uremia, and Parkinson’s disease. The exclusion criteria were: (1) inability to read or understand the questionnaire; (2) severe physical discomfort or other conditions preventing completion of the survey; and (3) severe hearing or speech impairment, acute delirium, or cognitive/communication problems that precluded reliable responses.

### 2.2 Data collection

Data were collected using a structured questionnaire administered in the outpatient chronic disease clinic. Eligible participants were approached by trained research staff, informed about the study purpose and procedures, and invited to participate. After providing informed consent, participants completed the questionnaire on-site. For participants with limited literacy or poor vision, trained research staff provided neutral assistance by reading the questionnaire items aloud and recording responses without interpretation. Completed questionnaires were checked immediately for missing or implausible responses, and participants were asked to clarify or complete missing responses whenever feasible.

The questionnaire consisted of two parts: (1) sociodemographic and clinical characteristics, including age, sex, place of residence, educational level, marital status, living arrangement, type of medical insurance, smoking and drinking status, personal monthly income, and number of chronic conditions; and (2) standardized measures of treatment burden, personal mastery, social support, and HRQoL.

### 2.3 Measures

#### 2.3.1 Sociodemographic and clinical characteristics

Sociodemographic and clinical characteristics were collected using a structured form and included age, sex, place of residence, educational level, marital status, living arrangement, type of medical insurance, smoking and drinking status, personal monthly income, and number of chronic conditions.

#### 2.3.2 Treatment burden

Treatment burden was assessed using the Treatment Burden Questionnaire (TBQ). The TBQ measures the workload imposed by healthcare and its impact on daily life, including domains such as medication management, self-monitoring, medical appointments, administrative tasks, lifestyle changes, financial burden, and the social impact of treatment (14). Each item is scored from 0 to 10, with higher total scores indicating greater treatment burden. The Chinese version of the TBQ has demonstrated acceptable psychometric properties in previous studies (15). In the present study, the internal consistency of the scale was acceptable, with a Cronbach’s *α* of 0.696.

#### 2.3.3 Personal mastery

Personal mastery was measured using the 7-item Personal Mastery Scale developed by Pearlin and Schooler (7). This scale assesses the extent to which individuals perceive themselves as having control over important life circumstances and being able to manage life demands effectively. Items are rated on a Likert-type scale, and negatively worded items are reverse-coded before summation, with higher scores indicating greater personal mastery. The Chinese version of the Mastery Scale has been psychometrically validated by Chen et al. (16), showing acceptable reliability and construct validity. In the present study, Cronbach’s *α* for the scale was 0.738.

#### 2.3.4 Social support

Social support was assessed using the Social Support Rating Scale (SSRS), a widely used instrument in Chinese populations. The SSRS contains 10 items and comprises three dimensions: objective support, subjective support, and support utilization. Higher total scores indicate greater perceived and received social support (17). The Chinese version of the SSRS has demonstrated good reliability and validity in previous studies. In the present study, Cronbach’s *α* for the total scale was 0.841.

#### 2.3.5 Health-related quality of life

Health-related quality of life (HRQoL) was assessed using the 12-item Short Form Health Survey, version 2 (SF-12v2) (18). The SF-12v2 covers eight domains of health, including general health, physical functioning, role limitations due to physical problems, bodily pain, vitality, social functioning, role limitations due to emotional problems, and mental health. Physical Component Summary (PCS) and Mental Component Summary (MCS) scores were derived according to the standard scoring procedure for the instrument, with higher scores indicating better HRQoL. The Chinese version of the SF-12v2 has demonstrated acceptable reliability and validity in older adults (19). In the present study, after coding all items in the same direction for reliability analysis, Cronbach’s *α* coefficients were 0.734 for the PCS-related item set and 0.728 for the MCS-related item set, indicating acceptable internal consistency.

### 2.4 Ethical considerations

The study protocol was reviewed and approved by the Institutional Review Board of Nanjing Drum Tower Hospital, the Affiliated Hospital of Nanjing University Medical School (Approval No. 2024-663-01). All participants provided informed consent prior to participation. Written informed consent was obtained whenever possible; when written consent was not feasible, verbal consent was obtained in accordance with the approved study procedures. The study was conducted in accordance with the Declaration of Helsinki. Trial registration was not applicable because this was an observational, non-interventional study.

### 2.5 Statistical analysis

Data were analyzed using IBM SPSS Statistics version 27.0 and the PROCESS macro version 5.0 (20). Continuous variables are presented as mean ± standard deviation (SD), and categorical variables are presented as frequencies and percentages.

Harman’s single-factor test was used to assess common method bias. Pearson correlation analysis was performed to examine the bivariate associations among treatment burden, personal mastery, social support, PCS, and MCS.

To examine the hypothesized statistical moderated mediation model, PROCESS Model 7 was used. Treatment burden was specified as the independent variable, personal mastery as the mediator, social support as the moderator of the association between treatment burden and personal mastery, and PCS or MCS as the dependent variable. Treatment burden and social support were mean-centered before creating the interaction term. Age, BMI, sex, income, and education were selected *a priori* as core covariates in the primary adjusted models because they are commonly associated with treatment burden, personal mastery, social support, and HRQoL, and were considered key potential confounders. Place of residence, marital status, living arrangement, type of medical insurance, smoking status, and drinking status were collected to describe the study population and to allow exploratory comparisons. To examine the robustness of the main findings, sensitivity analyses were further conducted by additionally adjusting for the number of chronic conditions and other collected sociodemographic and lifestyle variables, including place of residence, marital status, living arrangement, type of medical insurance, smoking status, and drinking status. Polypharmacy status was not included because the number of regularly used medications was not systematically collected. Bias-corrected bootstrapping with 5,000 resamples was used to estimate conditional indirect associations and the index of moderated mediation. An indirect association was considered statistically significant when the 95% bootstrap confidence interval did not include zero.

Given the cross-sectional design, the moderated mediation results were interpreted as statistical associations rather than evidence of temporal or causal pathways.

## 3 Results

### 3.1 Sociodemographic characteristics

Among the 470 participants, 350 (74.5%) were male and 120 (25.5%) were female. The mean age was 74.40 ± 8.96 years. The relatively high proportion of male participants may be related to the vascular specialty recruitment context of the outpatient clinic. Detailed sociodemographic and clinical characteristics of the participants are presented in Table 1.

**Table 1:** Characteristics of the study participants (*N* = 470).

Variable	Value
Age (years)	74.40 ± 8.96
BMI (kg/m^2^)	22.62 ± 3.64
Sex	
Male	350 (74.5)
Female	120 (25.5)
Ethnicity	
Han	450 (95.7)
Other ethnic groups	20 (4.3)
Smoking status	
Non-smoker	270 (57.4)
Smoker	200 (42.6)
Alcohol drinking status	
Non-drinker	400 (85.1)
Drinker	70 (14.9)
Number of chronic conditions	
2	160 (34.0)
3	130 (27.7)
≥4	180 (38.3)
Education level	
Primary school or below	150 (31.9)
Junior high school	140 (29.8)
Senior high school	100 (21.3)
Junior college	30 (6.4)
Bachelor’s degree	50 (10.6)
Place of residence	
Urban	320 (68.1)
Rural	150 (31.9)
Living arrangement	
Living alone	60 (12.8)
Living with spouse	230 (48.9)
Living with children	110 (23.4)
Living with spouse and children	70 (14.9)
Personal monthly income (CNY)	
≤2,000	100 (21.3)
2,001–4,000	90 (19.1)
4,001–6,000	220 (46.8)
≥6,001	60 (12.8)
Marital status	
Married	410 (87.2)
Widowed	50 (10.6)
Divorced	10 (2.1)
Type of medical insurance	
Urban–rural resident basic medical insurance	250 (53.2)
Urban employee basic medical insurance	210 (44.7)
Self-paid	10 (2.1)
Treatment burden (TB)	74.59 ± 10.04
Personal mastery (PM)	20.80 ± 2.92
Social support (SS)	35.59 ± 9.11
Physical component summary (PCS)	25.45 ± 8.47
Mental component summary (MCS)	41.14 ± 10.72

Continuous variables are presented as mean ± standard deviation; categorical variables are presented as *n* (%).

### 3.2 Common method bias

Harman’s single-factor test was used to assess common method bias. Eight factors had eigenvalues greater than 1, and the first factor accounted for 17.1% of the total variance, which was below the commonly used threshold of 40%. This result suggests that common method variance was unlikely to substantially bias the findings.

### 3.3 Correlations among treatment burden, personal mastery, social support, and HRQoL

Means, standard deviations, and Pearson correlation coefficients for the main study variables are shown in Table 2. Treatment burden was negatively correlated with personal mastery (*r* = −0.390, *p* < 0.001), social support (*r* = −0.624, *p* < 0.001), and PCS (*r* = −0.222, *p* < 0.001), but positively correlated with MCS (*r* = 0.305, *p* < 0.001). Personal mastery was positively correlated with social support (*r* = 0.214, *p* < 0.001) and PCS (*r* = 0.206, *p* < 0.001), but was not significantly correlated with MCS (*r* = −0.033, *p* = 0.471). Social support was not significantly correlated with PCS (*r* = 0.076, *p* = 0.101), but was negatively correlated with MCS (*r* = −0.238, *p* < 0.001). PCS was not significantly correlated with MCS (*r* = −0.044, *p* = 0.339).

**Table 2:** Means, standard deviations, and Pearson correlations among the study variables (*N* = 470).

Variable	M	SD	1	2	3	4	5
1. TB	74.59	10.04	–				
2. PM	20.80	2.92	−0.390***	–			
3. SS	35.59	9.11	−0.624***	0.214***	–		
4. PCS	25.45	8.47	−0.222***	0.206***	0.076	–	
5. MCS	41.14	10.72	0.305***	−0.033	−0.238***	−0.044	–

*p < 0.05, **p < 0.01, ***p < 0.001. Continuous variables are presented as mean ± standard deviation. Abbreviations: TB, treatment burden; PM, personal mastery; SS, social support; PCS, physical component summary; MCS, mental component summary.

### 3.4 Moderated mediation analysis for physical quality of life

Results of the adjusted moderated mediation model for PCS are presented in Table 3. After controlling for age, BMI, sex, income, and education, treatment burden was significantly negatively associated with personal mastery (*b* = −0.1185, SE = 0.0156, *p* < 0.001). The interaction between treatment burden and social support was also significant (*b* = 0.0056, SE = 0.0012, *p* < 0.001), indicating that social support moderated the association between treatment burden and personal mastery.

**Table 3:** Adjusted moderated mediation analyses for quality of life.

Panel A: Physical quality of life (PCS)						
Statistic	*b*/Effect	SE/BootSE	*t*	*p*	LLCI	ULCI
Mediator model (outcome = PM)						
TB	−0.1185	0.0156	−7.5825	<0.001	−0.1492	−0.0878
SS	−0.0099	0.0173	−0.5735	0.567	−0.0439	0.0240
TB × SS	0.0056	0.0012	4.8219	<0.001	0.0033	0.0079
Outcome model (outcome = PCS)						
TB (direct effect)	−0.1417	0.0411	−3.4435	<0.001	−0.2225	−0.0608
PM	0.4064	0.1424	2.8544	0.005	0.1266	0.6861
Indirect effect at low SS	−0.0688	0.0255			−0.1229	−0.0220
Indirect effect at moderate SS	−0.0482	0.0183			−0.0875	−0.0152
Indirect effect at high SS	−0.0275	0.0126			−0.0562	−0.0071
Index of moderated mediation	0.0023	0.0009			0.0006	0.0042

Panel B: Mental quality of life (MCS)						
Statistic	*b*/Effect	SE/BootSE	*t*	*p*	LLCI	ULCI
Outcome model (outcome = MCS)						
TB (direct effect)	0.3639	0.0510	7.1361	<0.001	0.2637	0.4642
PM	0.3894	0.1765	2.2065	0.028	0.0426	0.7362
Indirect effect at low SS	−0.0659	0.0317			−0.1308	−0.0067
Indirect effect at moderate SS	−0.0462	0.0221			−0.0916	−0.0048
Indirect effect at high SS	−0.0263	0.0140			−0.0569	−0.0025
Index of moderated mediation	0.0022	0.0011			0.0002	0.0047

All models were adjusted for age, BMI, sex, income, and education. Covariate coefficients are not shown for brevity. The mediator model (Outcome = PM) was identical for the PCS and MCS models and is presented once in Panel A. TB, treatment burden; PM, personal mastery; SS, social support; PCS, physical component summary; MCS, mental component summary; *b*, unstandardized coefficient; SE, standard error; BootSE, bootstrap standard error; CI, confidence interval.

As shown in Table 3, personal mastery was positively associated with PCS (*b* = 0.4064, SE = 0.1424, *p* = 0.005), whereas treatment burden remained directly and negatively associated with PCS (*b* = −0.1417, SE = 0.0411, *p* < 0.001). The conditional indirect associations of treatment burden with PCS through personal mastery were significant at low (effect = −0.0688, 95% CI [−0.1229, −0.0220]), moderate (effect = −0.0482, 95% CI [−0.0875, −0.0152]), and high (effect = −0.0275, 95% CI [−0.0562, −0.0071]) levels of social support. The index of moderated mediation was significant (index = 0.0023, BootSE = 0.0009, 95% CI [0.0006, 0.0042]), suggesting that the negative indirect association between treatment burden and PCS through personal mastery became weaker as social support increased.

**Figure 2:**
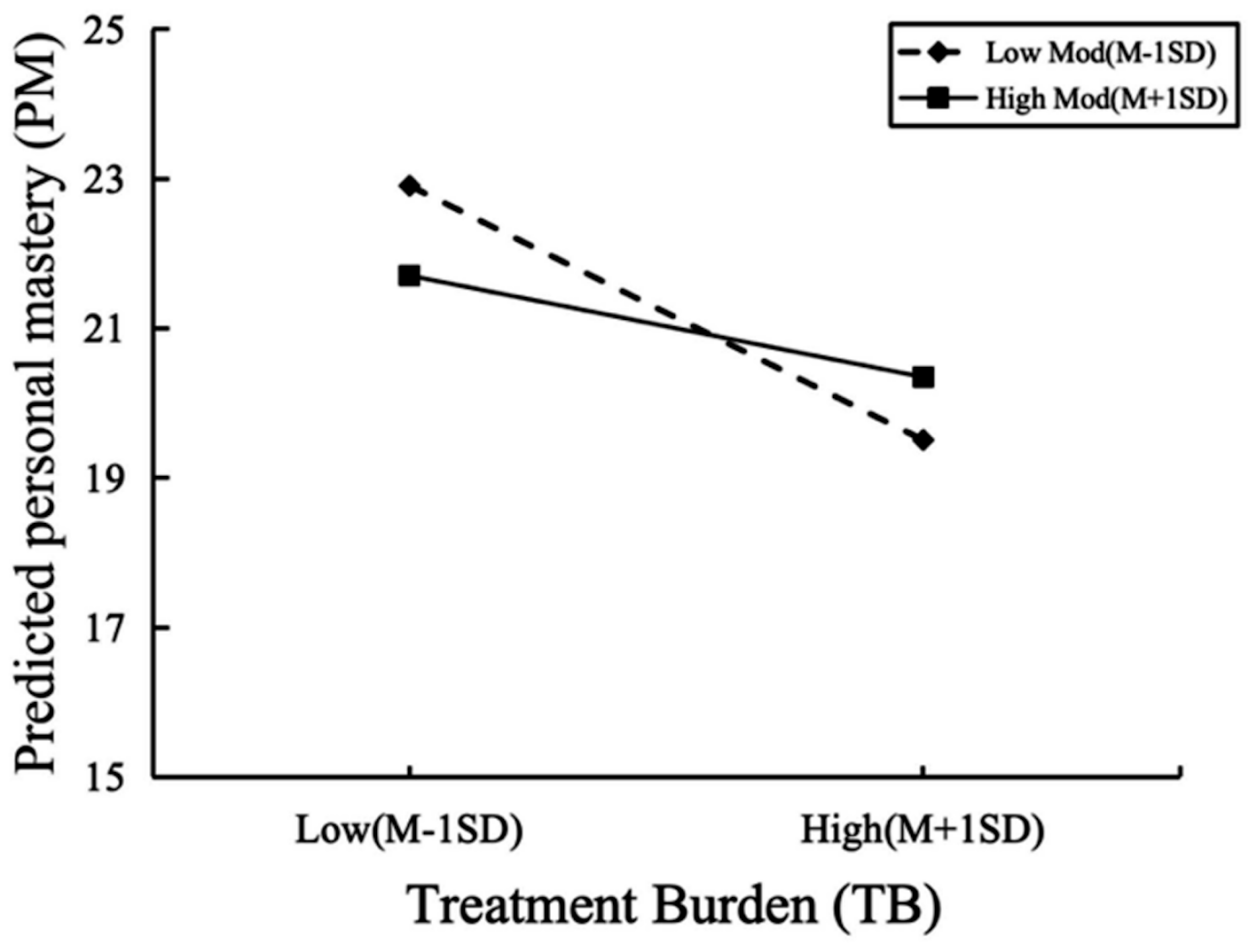
Simple slopes of the association between treatment burden and personal mastery at low and high levels of social support.

Simple slope analysis further showed that the negative association between treatment burden and personal mastery was strongest at low levels of social support (*b* = −0.1693, 95% CI [−0.2066, −0.1320], *p* < 0.001), weaker at moderate levels of social support (*b* = −0.1185, 95% CI [−0.1492, −0.0878], *p* < 0.001), and weakest at high levels of social support (*b* = −0.0676, 95% CI [−0.1044, −0.0308], *p* < 0.001). The interaction pattern is illustrated in Figure 2.

### 3.5 Moderated mediation analysis for mental quality of life

Results of the adjusted moderated mediation model for MCS are also presented in Table 3. Because social support moderated the same first-stage association from treatment burden to personal mastery, the mediator model was identical to that used in the PCS model. Treatment burden was significantly negatively associated with personal mastery (*b* = −0.1185, SE = 0.0156, *p* < 0.001), and the interaction between treatment burden and social support was significant (*b* = 0.0056, SE = 0.0012, *p* < 0.001).

In the outcome model, personal mastery was positively associated with MCS (*b* = 0.3894, SE = 0.1765, *p* = 0.028). The direct association between treatment burden and MCS was positive and significant (*b* = 0.3639, SE = 0.0510, *p* < 0.001), whereas the conditional indirect associations through personal mastery were negative. Specifically, the conditional indirect associations were significant at low (effect = −0.0659, 95% CI [−0.1308, −0.0067]), moderate (effect = −0.0462, 95% CI [−0.0916, −0.0048]), and high (effect = −0.0263, 95% CI [−0.0569, −0.0025]) levels of social support. The index of moderated mediation was significant (index = 0.0022, BootSE = 0.0011, 95% CI [0.0002, 0.0047]). These findings indicate an inconsistent moderated mediation pattern for MCS, in which treatment burden was negatively associated with MCS indirectly through lower personal mastery, while its direct association with MCS was positive.

Because the same first-stage moderated association from treatment burden to personal mastery was used in both the PCS and MCS models, the simple slope pattern was identical across the two models; therefore, only one interaction plot is presented in Figure 2.

### 3.6 Robustness of the findings

Overall, the adjusted moderated mediation models shown in Table 3 indicate that treatment burden was negatively associated with personal mastery, and that social support significantly moderated this association. The conditional indirect associations of treatment burden with both PCS and MCS through personal mastery were negative and statistically significant across low, moderate, and high levels of social support. The significant indices of moderated mediation further suggested that these negative indirect associations became weaker as social support increased.

Sensitivity analyses additionally adjusting for the number of chronic conditions, place of residence, marital status, living arrangement, type of medical insurance, smoking status, and drinking status yielded results consistent with the primary models. Treatment burden remained negatively associated with personal mastery, the interaction between treatment burden and social support remained significant, and personal mastery remained positively associated with both PCS and MCS. Exploratory sex-related analyses showed no clear evidence that the first-stage moderated association differed by sex. In the MCS model, the association between personal mastery and MCS appeared stronger among female participants in stratified analyses, but the corresponding interaction test did not provide definitive evidence of sex moderation. Sensitivity analyses are presented in Supplementary Table S1, and exploratory sex-related analyses are presented in Supplementary Tables S2, S3.

## 4 Discussion

This study examined the association between treatment burden and HRQoL in older adults with multimorbidity in Jiangsu Province, China, and further explored the statistical indirect role of personal mastery and the moderating role of social support. Three main findings emerged. First, greater treatment burden was associated with lower personal mastery, and lower personal mastery was associated with poorer HRQoL. Second, the negative association between treatment burden and personal mastery was weaker at higher levels of social support. Third, the pattern differed across outcome domains: for PCS, the results were consistent with a partial statistical moderated mediation model, whereas for MCS, the direct and indirect associations were in opposite directions, suggesting a more complex cross-sectional pattern. These findings extend previous research showing that multimorbidity-related treatment burden is associated with poorer patient-reported outcomes and suggest that this association may vary according to patients’ psychological and social resources (21).

One important contribution of this study is that it identified personal mastery as a plausible psychological correlate within the statistical indirect association between treatment burden and HRQoL. Treatment burden is increasingly understood as the workload generated by healthcare and its association with patients’ functioning and well-being, especially in people living with multimorbidity (22). Recent integrative evidence suggests that this burden includes not only medication-taking and appointment attendance, but also ongoing cognitive, emotional, financial, and relational work (5). For older adults, these demands may coincide with age-related changes in function, energy, and adaptive reserve, making them difficult to manage (23). Against this background, the observed indirect association through personal mastery is theoretically coherent. Personal mastery reflects the extent to which individuals feel able to influence important life circumstances, and stronger mastery has been associated with better physical, behavioral, and psychosocial outcomes in older adults (24). In the present cross-sectional data, older adults reporting greater treatment burden also tended to report lower personal mastery, which in turn was associated with poorer HRQoL. This pattern should be interpreted as a statistical association rather than evidence of a temporal or causal pathway (5, 25).

The findings for physical HRQoL were relatively straightforward. Treatment burden showed both a direct negative association with PCS and a negative indirect association through personal mastery. This suggests that physical HRQoL may be particularly sensitive to the combined presence of healthcare workload and perceived capacity to manage that workload. At the practical level, greater treatment burden may be accompanied by fatigue, disrupted routines, reduced activity, and greater difficulty with the daily demands of chronic disease management (26, 27). At the psychological level, lower personal mastery may be linked to poorer perceived physical functioning and lower physical quality of life. This interpretation is consistent with Chinese studies showing that multimorbidity is associated with poorer HRQoL among older adults and is also compatible with the broader treatment burden literature, which emphasizes the importance of the balance between care workload and patient capacity (26, 27). The fact that the PCS-related associations remained significant after adjustment for age, BMI, sex, income, and education suggests that these associations were not fully explained by the core sociodemographic covariates included in the model.

Another major finding of this study is that social support moderated the association between treatment burden and personal mastery. In the present study, the negative association between treatment burden and mastery was strongest under low social support and weakest under high social support, indicating that the association between treatment burden and personal mastery was attenuated among participants with higher social support. This pattern is consistent with the stress-buffering hypothesis, which proposes that social support may reduce the strength of the association between stressors and adverse psychological or health-related outcomes by providing emotional, instrumental, and informational resources (10). Several mechanisms may help explain this pattern. Instrumental support may reduce the day-to-day workload associated with treatment by assisting with transportation, appointment-related tasks, medication organization, and communication with healthcare providers (28, 29). Emotional support may be associated with less helplessness, frustration, and anticipatory stress, whereas informational support may help patients better understand treatment requirements and self-care demands (10). For older adults with multimorbidity, these forms of support may be linked to a stronger sense of competence and control despite substantial care demands. Evidence from Chinese older adults also suggests that informal social support is associated with better health and quality of life, which supports the plausibility of this interpretation in the local context (30). Importantly, our results suggest that social support was not merely associated with HRQoL in a general sense, but was related to the strength of the association between treatment burden and personal mastery. Older adults with high treatment burden and limited support may therefore represent a group requiring closer clinical attention.

The findings for mental HRQoL were more complex. Unlike the PCS model, the MCS model showed a negative indirect association through personal mastery but a positive direct association with treatment burden after adjustment. This pattern is better interpreted as an inconsistent statistical moderated mediation pattern rather than as a contradiction. The negative indirect association is theoretically plausible: greater treatment burden was associated with lower mastery, and lower mastery was associated with poorer mental HRQoL in the adjusted model. This is consistent with a broader literature linking perceived control to emotional well-being (24, 25). The more difficult issue is the positive direct association between treatment burden and MCS. One possible explanation is adaptation. The response-shift framework suggests that people living with chronic illness may recalibrate internal standards, reprioritize values, or reconceptualize quality of life as they adapt to illness (28). Under this view, some older adults with heavier treatment involvement may report relatively preserved mental quality-of-life appraisals because they may have adjusted their expectations, developed adaptive routines, or received more family and healthcare contact (31). Another possibility is that the positive direct association reflects processes not captured in the current model, such as treatment engagement, symptom vigilance, perceived safety from being under care, or increased contact with supportive others (28–30). A third possibility is statistical suppression, such that once mastery and key covariates are controlled, a previously obscured positive component of treatment engagement becomes visible. This may help explain why treatment burden was positively correlated with MCS at the bivariate level, whereas the decomposed model revealed a negative indirect association through personal mastery alongside a positive direct association. Overall, mental HRQoL in older adults with multimorbidity may reflect a complex mix of burden, adaptation, support, and meaning-making (31).

The clinical setting of the present study should also be considered when interpreting these findings. China is experiencing rapid population ageing, and multimorbidity has become increasingly common among older adults. Chinese studies have shown that multimorbidity is associated with poorer HRQoL, while recent evidence suggests that greater treatment burden in older Chinese adults is associated with lower chronic disease self-efficacy (23, 26). Although self-efficacy and personal mastery are not identical, both tap into perceived capacity to manage illness-related demands. Our findings therefore extend emerging Chinese evidence by suggesting that treatment burden is associated not only with self-management confidence, but also with broader physical and mental HRQoL, and that these associations may vary according to the social support available to patients (23, 24). Because this study was conducted in Jiangsu Province, an economically developed region with relatively strong healthcare resources, the findings are noteworthy. Even in a setting with comparatively good access to tertiary care, treatment workload was still associated with lower mastery and poorer physical HRQoL. This suggests that the challenge is not simply a matter of service availability, but also involves the cumulative workload of navigating chronic care over time (23, 26).

These findings have several practical implications. First, treatment burden should be considered an important clinical assessment focus in older adults with multimorbidity. Simplifying medication regimens where appropriate, reducing avoidable care demands, improving coordination across providers, and aligning treatment plans with patient capacity may help reduce treatment workload and support HRQoL (32). Second, mastery-related support may be valuable in clinical care. Approaches such as collaborative goal setting, self-management education, problem-solving support, and nurse-led coaching may help older adults maintain a sense of control when managing complex treatment demands (33). Third, the moderating role of social support suggests that clinicians should routinely assess family and community support among older adults with multimorbidity and consider mobilizing these resources when support is limited (34). In this sense, supporting HRQoL may require attention not only to healthcare coverage and service availability, but also to treatment fit, care coordination, patient capacity, and the social conditions that support everyday self-management (11, 26).

This study has several limitations. First, the cross-sectional design precludes causal inference, and the moderated mediation results should be interpreted as statistical associations rather than temporal or causal pathways. Second, participants were recruited from a single tertiary hospital, which may limit the generalizability of the findings to other settings and populations. Third, the sample included a relatively high proportion of men, which may reflect the vascular specialty recruitment context but may limit the generalizability of the findings to older women with multimorbidity. The exploratory sex-related analyses suggested that the overall first-stage moderated association did not clearly differ by sex. Although the association between personal mastery and MCS appeared stronger among female participants in stratified analyses, the corresponding interaction test did not provide definitive evidence of sex moderation. These findings should therefore be interpreted cautiously because the number of female participants was relatively limited and the analyses were exploratory. Future studies with more balanced sex distributions are needed to confirm whether sex modifies the role of personal mastery in the association between treatment burden and mental HRQoL. Fourth, although the number of chronic conditions and several sociodemographic and lifestyle variables were included in sensitivity analyses, other important clinical factors, such as detailed disease types in the adjusted models, disease severity, polypharmacy, treatment complexity, hospitalization history, and formal cognitive status, were not systematically measured and may have influenced the observed associations.

## 5 Conclusion

In conclusion, this cross-sectional study found that treatment burden was associated with poorer physical HRQoL among older adults with multimorbidity and showed negative indirect associations with both physical and mental HRQoL through lower personal mastery. These negative indirect associations were weaker at higher levels of social support. The pattern was clearer for physical HRQoL and more complex for mental HRQoL, where the direct and indirect associations were in opposite directions. These findings highlight the relevance of treatment workload, personal mastery, and social support when considering strategies to support HRQoL in older adults with multimorbidity. Longitudinal and intervention studies are needed to clarify the temporal and causal nature of these associations.

## Data Availability

The raw data supporting the conclusions of this article will be made available by the authors on reasonable request.
