# Supplementary material for: The association between treatment burden and quality of life in older adults with multimorbidity: the mediating role of personal mastery and the moderating role of social support—a cross-sectional study in China
Source: Front Public Health. 2026 Aug 3;14:1859774. doi: 10.3389/fpubh.2026.1859774 (PMC13477879; doi:10.3389/fpubh.2026.1859774)
Supplement: Supplementary file 1 [file Table_1.docx]

**Supplementary Tables**

**Supplementary Table S1. Sensitivity analyses additionally adjusting for clinical, sociodemographic, and lifestyle variables**

| **Model** | **Statistic** | **b** | **SE** | **t** | **p** | **LLCI** | **ULCI** |
| --- | --- | --- | --- | --- | --- | --- | --- |
| Mediator model (Outcome = PM) | TB | -0.1144 | 0.0158 | -7.2157 | <0.001 | -0.1455 | -0.0832 |
| Mediator model (Outcome = PM) | SS | -0.0078 | 0.0174 | -0.4464 | 0.655 | -0.0420 | 0.0265 |
| Mediator model (Outcome = PM) | TB × SS | 0.0054 | 0.0012 | 4.6421 | <0.001 | 0.0031 | 0.0077 |
| Outcome model (Outcome = PCS) | TB | -0.1451 | 0.0414 | -3.5085 | <0.001 | -0.2264 | -0.0638 |
| Outcome model (Outcome = PCS) | PM | 0.4244 | 0.1430 | 2.9688 | 0.003 | 0.1435 | 0.7054 |
| Outcome model (Outcome = MCS) | TB | 0.3539 | 0.0519 | 6.8188 | <0.001 | 0.2519 | 0.4558 |
| Outcome model (Outcome = MCS) | PM | 0.4207 | 0.1793 | 2.3460 | 0.019 | 0.0683 | 0.7731 |

*Note.* Sensitivity models additionally adjusted for number of chronic conditions, place of residence, marital status, living arrangement, type of medical insurance, smoking status, and drinking status, in addition to age, BMI, sex, income, and education. TB, treatment burden; PM, personal mastery; SS, social support; PCS, physical component summary; MCS, mental component summary; CI, confidence interval.
