# Supplementary material for: The association between treatment burden and quality of life in older adults with multimorbidity: the mediating role of personal mastery and the moderating role of social support—a cross-sectional study in China
Source: Front Public Health. 2026 Aug 3;14:1859774. doi: 10.3389/fpubh.2026.1859774 (PMC13477879; doi:10.3389/fpubh.2026.1859774)
Supplement: Supplementary file 2 [file Table_2.docx]

**Supplementary Tables**

**Supplementary Table S2. Exploratory sex-related interaction analyses**

| **Model** | **Statistic** | **b** | **SE** | **t** | **p** | **LLCI** | **ULCI** |
| --- | --- | --- | --- | --- | --- | --- | --- |
| Mediator model | Sex (male vs female) | 0.1067 | 0.3232 | 0.3301 | 0.741 | -0.5284 | 0.7417 |
| Mediator model | TB × SS | 0.0071 | 0.0020 | 3.6435 | <0.001 | 0.0033 | 0.0110 |
| Mediator model | TB × Sex | 0.0542 | 0.0379 | 1.4279 | 0.154 | -0.0204 | 0.1288 |
| Mediator model | SS × Sex | 0.0460 | 0.0401 | 1.1464 | 0.252 | -0.0329 | 0.1249 |
| Mediator model | TB × SS × Sex | -0.0023 | 0.0025 | -0.9506 | 0.342 | -0.0072 | 0.0025 |
| PCS model | Sex (male vs female) | 2.2723 | 6.4609 | 0.3517 | 0.725 | -10.4242 | 14.9689 |
| PCS model | TB × Sex | 0.1506 | 0.0944 | 1.5945 | 0.112 | -0.0350 | 0.3362 |
| PCS model | PM × Sex | -0.1357 | 0.3099 | -0.4378 | 0.662 | -0.7446 | 0.4733 |
| MCS model | Sex (male vs female) | 13.6621 | 8.0075 | 1.7062 | 0.089 | -2.0737 | 29.3979 |
| MCS model | TB × Sex | -0.0132 | 0.1171 | -0.1129 | 0.910 | -0.2432 | 0.2168 |
| MCS model | PM × Sex | -0.7312 | 0.3840 | -1.9038 | 0.058 | -1.4859 | 0.0235 |

*Note.* Female participants were the reference group. These analyses were exploratory and should be interpreted cautiously because the number of female participants was relatively limited. TB, treatment burden; PM, personal mastery; SS, social support; PCS, physical component summary; MCS, mental component summary; CI, confidence interval.
