# Supplementary material for: The association between treatment burden and quality of life in older adults with multimorbidity: the mediating role of personal mastery and the moderating role of social support—a cross-sectional study in China
Source: Front Public Health. 2026 Aug 3;14:1859774. doi: 10.3389/fpubh.2026.1859774 (PMC13477879; doi:10.3389/fpubh.2026.1859774)
Supplement: Supplementary file 3 [file Table_3.docx]

**Supplementary Tables**

**Supplementary Table S3. Sex-stratified conditional indirect associations from PROCESS Model 7**

| **Sex** | **Outcome** | **Effect** | **Estimate** | **BootSE** | **LLCI** | **ULCI** |
| --- | --- | --- | --- | --- | --- | --- |
| Female | PCS | Indirect effect at low SS | -0.1528 | 0.0737 | -0.3079 | -0.0236 |
| Female | PCS | Indirect effect at moderate SS | -0.1066 | 0.0543 | -0.2244 | -0.0156 |
| Female | PCS | Indirect effect at high SS | -0.0603 | 0.0389 | -0.1503 | -0.0042 |
| Female | PCS | Index of moderated mediation | 0.0046 | 0.0023 | 0.0006 | 0.0093 |
| Female | MCS | Indirect effect at low SS | -0.2127 | 0.0787 | -0.3648 | -0.0606 |
| Female | MCS | Indirect effect at moderate SS | -0.1483 | 0.0573 | -0.2679 | -0.0417 |
| Female | MCS | Indirect effect at high SS | -0.0840 | 0.0432 | -0.1802 | -0.0132 |
| Female | MCS | Index of moderated mediation | 0.0064 | 0.0027 | 0.0014 | 0.0118 |
| Male | PCS | Indirect effect at low SS | -0.0496 | 0.0239 | -0.0948 | -0.0021 |
| Male | PCS | Indirect effect at moderate SS | -0.0356 | 0.0175 | -0.0699 | -0.0016 |
| Male | PCS | Indirect effect at high SS | -0.0215 | 0.0128 | -0.0502 | -0.0004 |
| Male | PCS | Index of moderated mediation | 0.0016 | 0.0009 | 0.0000 | 0.0035 |
| Male | MCS | Indirect effect at low SS | -0.0261 | 0.0330 | -0.0967 | 0.0327 |
| Male | MCS | Indirect effect at moderate SS | -0.0187 | 0.0236 | -0.0683 | 0.0240 |
| Male | MCS | Indirect effect at high SS | -0.0113 | 0.0150 | -0.0443 | 0.0155 |
| Male | MCS | Index of moderated mediation | 0.0008 | 0.0011 | -0.0010 | 0.0035 |

*Note.* Conditional indirect associations and indices of moderated mediation were estimated using bias-corrected bootstrapping with 5,000 resamples. Models were adjusted for age, BMI, income, and education within each sex subgroup. BootSE, bootstrap standard error; CI, confidence interval.
